# Self-harm emergency department visits in Canada during the COVID-19 pandemic: evidence from a sentinel surveillance system

**DOI:** 10.1186/s40621-022-00411-8

**Published:** 2023-01-05

**Authors:** Shikha Saxena, Li Liu, Nathaniel Pollock, Steven R. McFaull

**Affiliations:** 1 Public Health Agency of Canada, Ottawa, ON Canada; 2 School of Arctic and Subarctic Studies, Labrador Campus, Memorial University, Happy Valley-Goose Bay, NL Canada

**Keywords:** Self-inflict, Surveillance, Injury, Intentional, Emergency department

## Abstract

**Background:**

Self-harm is a leading cause of morbidity and mortality globally, though the prevalence tends to be highest among adolescents. As an indicator in suicide surveillance, the incidence of self-harm is useful because it is sensitive to social, environmental, and economic conditions. During the COVID-19 pandemic, the epidemiology of self-harm has varied across contexts. This study aims to investigate the changes in self-harm emergency department visits in 2020 compared to a pre-pandemic period in 2018–2019.

**Methods:**

Self-harm emergency department visits were extracted from the Canadian Hospitals Injury Reporting and Prevention Program database from 2018 to 2020. We compared the data in 2020 with the pre-pandemic baseline in 2018–2019. We examined the changes in volume, the percentages of self-harm cases among all intentional injuries by sex, age group, and the proportions of self-harm cases by method of injury. We also quantified the time trends of the percentages of self-harm cases among all intentional injuries using Joinpoint regression.

**Results:**

The overall volume of emergency visits related to self-harm was higher in 2020 during weeks 24–51 compared to the average volumes for the same weeks of 2018–2019. Percentage of self-harm among all intentional injury emergency department visits was significantly higher by 6.1% among females (*p* < 0.05) and by 5.3% among males in 2020 than in 2018–2019 (*p* < 0.05). The 11-to-18-year age group showed an increase in the percentage of self-harm among all intentional injury emergency department visits by 7.4% in 2020 when compared to 2018–2019. Time trend analyses showed that the percentages of self-harm among all intentional injury emergency department visits were higher during weeks 4–52 in 2020 than in 2018–2019, for both males and females.

**Conclusions:**

The percentage of emergency department visits related to self-harm among all intentional injury visits were higher during 2020 than in 2018–2019. These results underscore the importance of continued surveillance of self-harm in Canada to better understand the sociodemographic factors affecting self-harm and to inform the prevention strategies and policies.

## Background

Globally, self-harm is a leading cause of morbidity and mortality (World Health Organization 2012, 2014; Vigo et al. 2020). Self-harm includes acts of self-poisoning or self-injury resulting in fatal or non-fatal outcomes (National Collaborating Centre for Mental Health 2004; Stewart et al. 2017). In Canada, self-harm is among the largest relative causes of disability-adjusted life years among non-communicable disease groups (Vigo et al. 2020). In the context of a public health approach to suicide prevention, the epidemiology of self-harm is important for surveillance because it is sensitive to changes in social, environmental, and economic conditions (Skinner et al. 2017). Preventing self-harm and providing care for people who experience self-harm-related injuries is a public health and health system priority in Canada (Jessula et al. 2022).

The prevalence of self-harm has varied over time and across contexts, with a global trend of higher prevalence among adolescents (Gillies et al. 2018; Lim et al. 2019). Data from the USA showed that the total number of emergency department (ED) visits for self-harm was stable between 2006 and 2013; however, the rate was highest in the 15–19 age group (Canner et al. 2018). In Ireland, rates of self-harm ED visits increased by 22% between 2007 and 2016 among 10–24 year old age group, with a pronounced increase for females (Griffin et al. 2018). The Multicentre Study of Self-harm in England showed a decrease in rates of self-harm ED visits among men and women from 2003 to 2006, and an increase from 2007 to 2012 (Clements et al. 2016). In Canada, self-harm hospitalizations declined from 1994 to 2014, though large sex differences were evident, as the rate among females aged 15–19 years old was 3.5 times higher than among males (Skinner et al. 2016). Substance use-related self-harm ED visits also increased in Canada from 2011 to 2019 (Campeau et al. 2022). In Ontario, self-harm ED visits among adolescents increased by 135% from 2009 to 2017 (Gardner et al. 2019).

Since the onset of the COVID-19 pandemic, the effects of COVID-19 on suicide and injuries associated with self-harm have been well investigated (Hawton et al. 2021; Holmes et al. 2020). Data from the Survey on COVID-19 and Mental Health in Canada showed that the prevalence of recent suicidal ideation increased from 2.7% in 2019 to 4.2% in fall 2021 (Liu et al. 2022). Administrative data from Canada revealed a 7% drop in the volume of self-harm ED visits for the period March 2020 to June 2021 in comparison with 2019 (Canadian Institute for Health Information 2021). This pattern varied by age and sex; females aged 10–24 years showed a 10% increase in the volume of self-harm ED visits from October 2020 to June 2021 (Canadian Institute for Health Information 2021). However, there might be a disparity between the visits captured by data from the Canadian Hospitals Injury Reporting and Prevention Program (CHIRPP) and those recorded in hospital administrative data (Johnson et al. 2019). A previous study found that data from CHIRPP captured more self-harm-related visits than hospital administrative data for the age group 6 to 17.99 years (Johnson et al. 2019).

To support a public health approach to prevent suicides and injuries associated with self-harm, ongoing surveillance of self-harm is needed to support policy and programming efforts. Using data from CHIRPP, the objectives of this study were to: (1) investigate changes in the volume of self-harm ED visits during the pandemic in 2020 compared to data from a pre-pandemic baseline period (2018–2019 average); (2) estimate self-harm ED visits as a percentage of all intentional cases by sex, age group, and distribution by method of injury in 2020 compared to 2018–2019; and (3) investigate the time trends in self-harm ED visits as a percentage of all intentional injury cases in 2020 compared to 2018–2019.

## Methods

### Data source

For this cross-sectional study, we used data from the Canadian Hospitals Injury Reporting and Prevention Program (CHIRPP). CHIRPP is an ED-based sentinel surveillance system launched in 1990; the system transitioned to an electronic format in 2011 (Public Health Agency of Canada 2020). The system involves a collaboration between the Public Health Agency of Canada, eleven pediatric hospitals, and nine general hospitals in Canada (Public Health Agency of Canada 2020). CHIRPP was not created to produce nationally representative incidence estimates of specific types of injuries and poisonings. CHIRPP collects data about patients that visit the ED due to an injury or poisoning, including information on the factors and mechanisms surrounding such injuries. For each visit, details about how the injury occurred are provided by the patient or accompanying caregiver, and clinical information is recorded by the attending physician on a CHIRPP form. Additional details about CHIRPP, including data quality procedures, have been reported elsewhere (Crain et al. 2016).

### Study population

ED visits by patients aged 5 and older were eligible for inclusion in our analysis. We used this lower age boundary to be consistent with other studies on self-harm, suicide attempts, and suicide deaths among pediatric populations (Burstein et al. 2019; Ruch et al. 2021). Records with missing date of birth were excluded from the data extraction.

### Case selection

The primary outcome for this study was ED visits related to self-harm injuries. We searched the CHIRPP database for self-harm-related visits on May 12, 2022. All records with the injury date between January 1, 2018, and December 31, 2020, were identified using either of the two variables, intent of injury or narrative description of the injury.

The *intent of injury* variable includes codes for ED visits related to intentional (self-harm, violence, assault, and child maltreatment) and unintentional (accidental) injuries. This was used to identify intentional injury and self-harm ED visits. Some visits related to self-harm were incorrectly coded as unintentional cases (Campeau et al. 2022), though details about the case were reported in the free-text field, *narrative description of the injury,* in the database. We identified additional self-harm-related injuries in the narrative descriptions related to self-inflicted injury, non-suicidal self-injury, self-mutilation, self-burning, suicide attempts, and suicide and then extracted all self-harm-related visits for analysis.

**Fig. 1:**
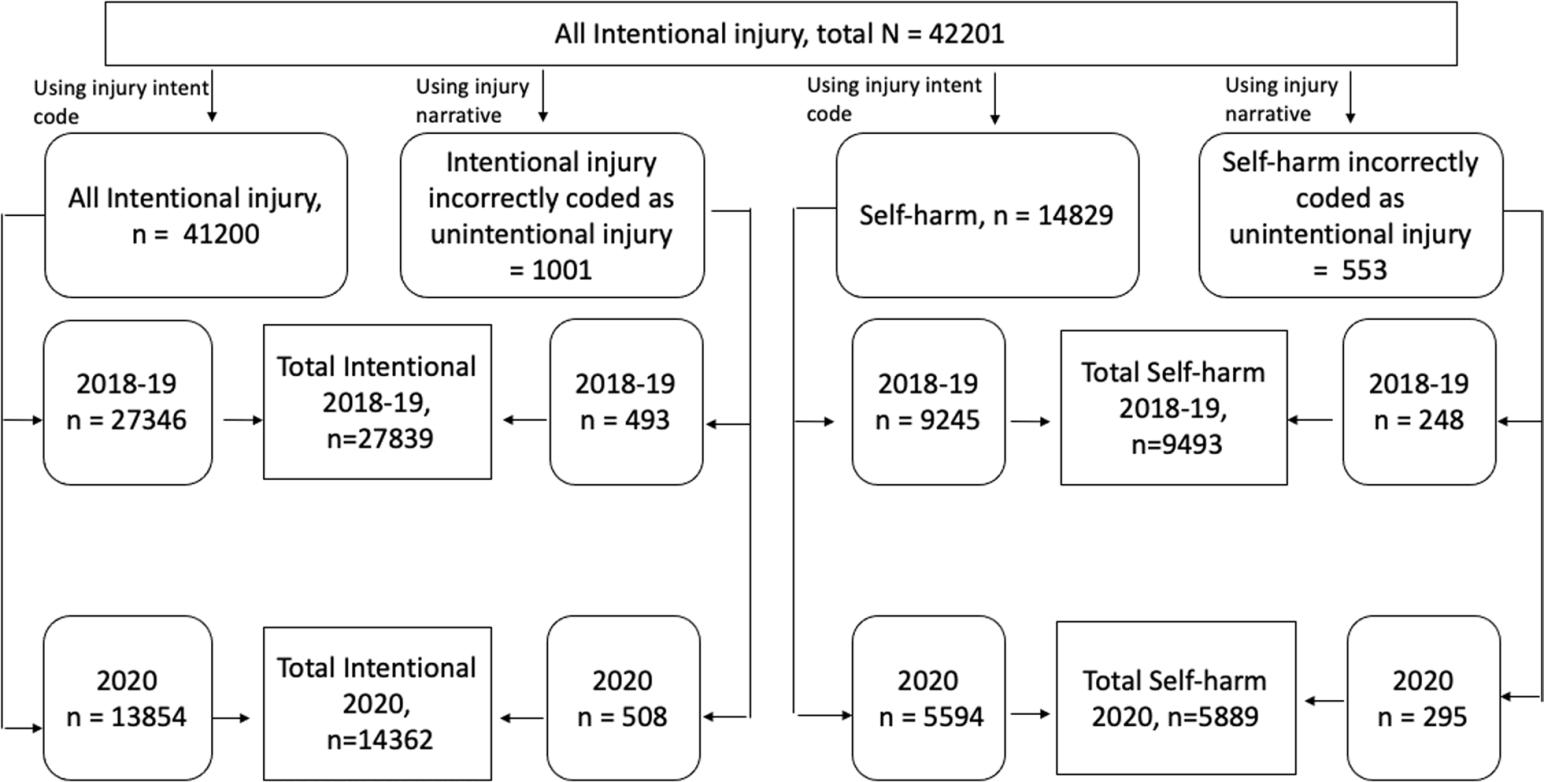
Case selection of self-harm and all intentional injuries in Canadian Hospitals Injury Reporting and Prevention Program, 2018–2019 and 2020

We conducted text comparisons of the narrative description of the injury for all unintentional cases using the keywords found in text fields of the confirmed self-harm injury cases. The keyword search was followed by manual identification and confirmation of cases by reading through each narrative. The keywords included French and English terms such as “suicide,” “ideation,” “anxiety,” or “ingestion” and variations thereof, and the comparisons were conducted using SAS 9.4 and Microsoft Excel 2016. Figure 1 shows the process for identifying self-harm-related visits.

### Data analyses

#### ED visit volumes

The percent changes of ED visit volumes for self-harm, all intentional injuries, and all ED cases in 2020 in comparison with the weekly average volumes of 2018–2019 were computed. The pre-pandemic cutoff was set at week 11 of 2020 (mid of March) as that aligns with the time when COVID-19-related public health measures were first introduced in Canada (Canadian Institute for Health Information 2022). Week 52 was excluded due to the variable number of days (2–5) in that week over the 3-year period (2018–2020).

#### Descriptive analyses

To describe self-harm ED visits, data from 2020 and 2018–2019 were compared by age, sex, and methods of injuries. To examine the distribution by sex and age group (5–10, 11–18, 19–29, 30–64, 65, and above), self-harm ED visits were calculated as a percentage of all intentional injuries ED visits for 2018–2019 and 2020 using SAS® 9.4. We used all intentional injuries as the denominator to calculate percentages of self-harm to avoid the impact of the volume changes in unintentional injuries during the pandemic. During the early part of the pandemic (March 2020 to May 2020), significantly fewer ED visits related to unintentional injuries were reported for children and adolescents (Keays et al. 2020). Similarly, a significant decrease was reported in the ED visits related to accidental fall injuries among all age groups between March 2020 and September 2020 (Canadian Institute for Health Information 2021). Since unintentional injuries constitute a majority of cases in CHIRPP, including them as a denominator may have “diluted” any effect.

To examine the distribution of self-harm ED visits by methods of injuries, percentage of self-harm ED visits for each method of injury among all self-harm ED visits were computed. The methods used for self-harm included four categories—poisoning, cut, no injury detected, and others. No injury detected included those who presented to the ED with thoughts of self-harm or suicidal ideation, and no injury was detected. Others included burns, asphyxiation, ingestion of foreign body, electric injury, sprains/strains, fractures, head injury, drowning, and injury to specific body structures (muscle, blood vessel, nerve) or internal organs. Pearson’s chi-square test with 95% confidence intervals was used to compare percentage of self-harm ED visits for the years 2020 and 2018–2019.

#### Trend analyses

Joinpoint Regression Program was used to compare weekly percentages of self-harm cases among all intentional injury cases in 2020 with the average weekly percentages in 2018–2019 by sex (National Cancer Institute 2022). Joinpoint Regression Program (version 4.9.0.0) detects inflection points and determines whether weekly percent changes (WPC) of segments are significantly different from zero (alpha = 0.05), with 95% confidence intervals (National Cancer Institute 2022).

## Results

### ED visit volumes

**Fig. 2:**
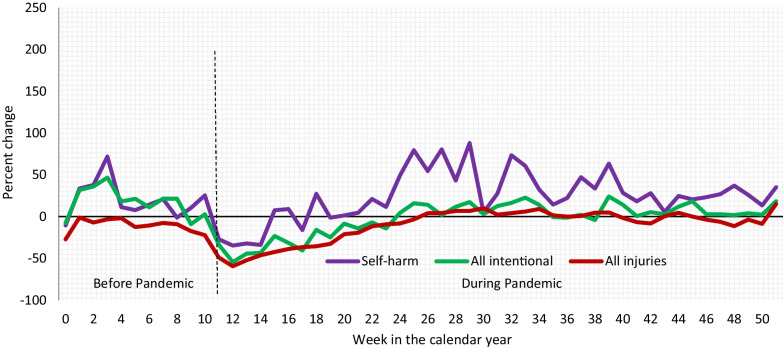
Percent change in volume of emergency department visits comparing 2020 to 2018–2019, both sexes, Canadian Hospitals Injury Reporting and Prevention Program (CHIRPP). Pandemic period between weeks 11 and 51 as indicated by dotted vertical line

The overall volume of self-harm ED visits was higher in 2020 during weeks 24–51 compared to the average volumes for the same weeks of 2018–2019 (Fig. 2). Volumes for all intentional and all ED visits showed a decrease for the first 12 weeks after pandemic (weeks 11–23); however, not much change was seen in their volumes from weeks 24–51 in 2020 compared to 2018–2019.

### Descriptive analyses

**Fig. 3:**
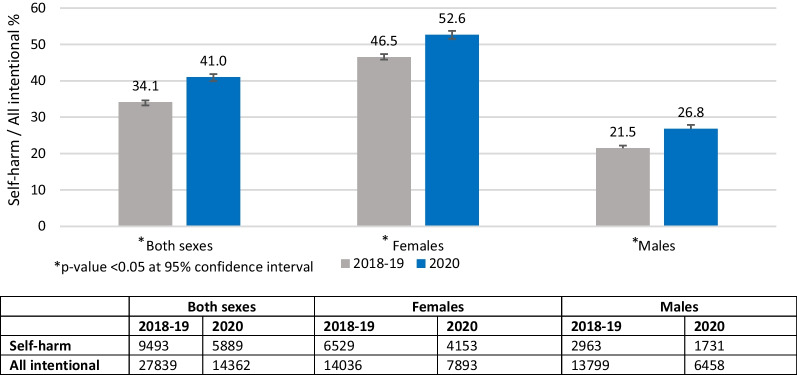
Percentage of self-harm among all intentional injury emergency department visits comparing 2020 to 2018–2019, by sex, Canadian Hospitals Injury Reporting and Prevention Program (CHIRPP). Missing sex for self-harm cases in 2018–2019, *n* = 1. Missing sex for self-harm cases in 2020, *n* = 5. Missing sex for all intentional cases in 2018–2019, *n* = 4. Missing sex for all intentional cases in 2020, *n* = 11

**Fig. 4:**
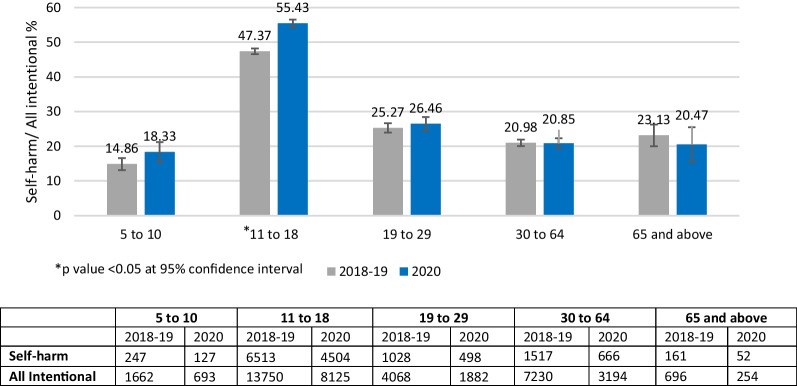
Percentage of self-harm among all intentional injury emergency department visits comparing 2020 to 2018–2019, by age groups, both sexes, Canadian Hospitals Injury Reporting and Prevention Program (CHIRPP)

A total of 5889 self-harm cases (41% of all intentional cases) were reported to CHIRPP in 2020. A total of 9493 self-harm cases (4843 in 2018 and 4650 in 2019) (34.1% of all intentional cases) were reported in 2018–2019 (see Fig. 1). Figure 3 shows the percentages of self-harm among all intentional injury ED visits for both sexes, males and females. Females had a higher percentage of self-harm ED visits than males for both 2020 and 2018–2019. Significant increase in the percentage of self-harm ED visits was seen in all groups during 2020 when compared to 2018–2019. Percentage of self-harm ED visits were 6.1% higher for females and 5.3% higher in males in 2020 than in 2018–2019. Figure 4 shows the distribution of the percentage of self-harm cases out of all intentional injury cases by age group. A significant increase in the percentage of self-harm visits was observed for the age group 11–18 years in 2020, whereas no significant differences were found for other ages. Table 1 shows the self-harm ED visits among males and females as a percentage of self-harm ED visits across each age group. Age group 11 to 18 years showed the maximum difference in the percentages of self-harm ED visits among males and females, where females accounted for > 75% of the self-harm ED visits both in 2018–2019 and 2020.

**Table 1:** Percentage of self-harm emergency department visits by sex and age for 2018–2019 and 2020, Canadian Hospitals Injury Reporting and Prevention Program

Age groups	2018–2019				2020			
	Males *N* (%)	Females *N* (%)	Total *N* (%)	*p* value	Males *N* (%)	Females *N* (%)	Total *N* (%)	*p* value
5 to 10	141 (57.1)	106 (42.9)	247 (100)	< 0.0001*	73 (57.5)	54 (42.5)	127 (100)	< 0.0001*
11 to 18**	1558 (23.9)	4955 (76.1)	6513 (100)		1023 (22.7)	3478 (77.3)	4501 (100)	
19 to 29	392 (38.1)	636 (61.9)	1028 (100)		223 (44.8)	275 (55.2)	498 (100)	
30 to 64	825 (54.4)	692 (45.6)	1517 (100)		364 (54.7)	302 (45.3)	666 (100)	
65 and above	100 (62.1)	61 (37.9)	161 (100)		30 (57.7)	22 (42.3)	52 (100)	

*Chi-sq *p* value < 0.05 at 95% confidence interval; **missing sex for 2020, *n* = 3

### Method of injury

Table 2 shows the percentage of self-harm ED visits for each method of injuries associated with self-harm in 2020 compared to 2018–2019. The percentage of self-harm cases showed a slight decrease in the percentage of self-harm due to cuts, and a slight increase due to poisoning in 2020 compared to 2018–2019.

**Table 2:** Percentage of self-harm emergency department visits by method of injury comparing 2020 to 2018–2019, all age groups, both sexes, Canadian Hospitals Injury Reporting and Prevention Program

	2018–2019 (*N* = 9493)		2020 (*N* = 5889)		p-value
	*n*	% (95% CI)	*n*	% (95% CI)	
Poisoning	4655	49.0 (48.0, 50.0)	3028	51.4 (50.1, 52.7)	0.01*
Cuts	2011	21.1 (20.4, 22.0)	1141	19.4 (18.4, 20.4)	
No injury reported	1697	17.9 (17.1, 18.7)	1022	17.4 (16.4, 18.3)	
Other	1130	11.9 (11.3, 12.6)	698	11.9 (11.0, 12.7)	

*Chi-sq *p* value < 0.05 at 95% confidence interval

### Time trend analyses

**Fig. 5:**
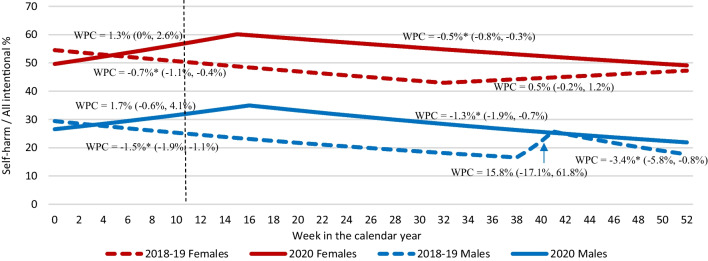
Time trend of self-harm among all intentional injury emergency department visits comparing 2020 to 2018–2019, by sex, all ages, Canadian Hospitals Injury Reporting and Prevention Program. Pandemic period between weeks 11 and 51 as indicated by dotted vertical line. WPC—weekly percent change; *represents significantly different from zero at the α = 0.05 level

Figure 5 shows the results of the Joinpoint regression analysis for the weekly percentages of self-harm out of all intentional injuries in 2020 in comparison with the weekly average percentages of 2018–2019 for males and females. Percentages of self-harm visits were higher during weeks 4–52 in 2020 than in 2018–2019, for both males and females. A significant decline in percentage of self-harm visits was seen in 2018–2019 (weeks 0–32 for females; weeks 0–38 for males), followed by a non-significant increase. However, both males and females showed a non-significant increase in the percentage of self-harm until week 16 in 2020, followed by a significant decrease between weeks 16–52.

## Discussion

This study examined trends in self-harm-related ED visits before and during the COVID-19 pandemic using a Canadian sentinel surveillance system. The ED visit volumes showed an increase percentage for self-harm during weeks 24–51 in 2020, a pandemic period, compared to 2018–2019 (Fig. 2). Among all intentional ED visits, self-harm visits increased by 5.3% for males, and 6.1% for females in 2020 compared to 2018–2019 (Fig. 3). Age group-specific comparisons revealed a significant increase of 7.4% for self-harm ED visits for ages 11 to 18 years in 2020 compared to 2018–2019 (Fig. 4). There was a slight decrease in injuries related to cuts and a slight increase in injuries related to poisoning in 2020 compared to 2018–2019 (Table 2). Soon after the implementation of first public health measures (week 11) in 2020 (Government of Canada 2021), a significant decline in the percentage of self-harm ED visits was seen during weeks 16–52 for both males and females (Fig. 5). However, trend analyses showed overall weekly percentages of self-harm to be higher in 2020 compared to 2018–2019 for both males and females.

The current evidence about the influence of COVID-19 pandemic on self-harm ED visits and hospitalizations and ED visits in Canada is limited. Data from the Canadian Institute of Health Information showed a decrease of 7% in volume of self-harm ED visits among ED visits for the period March 2020 to June 2021 compared with 2019 (Canadian Institute for Health Information 2021). This data showed larger decreases in the volume of self-harm ED visits in the first few months (Canadian Institute for Health Information 2021), which is consistent with our study. However, the percent change in volume of self-harm-related ED visits in our study was higher for most weeks later (weeks 24–51). This higher percent change in the volume of self-harm-related ED visits in our study should be interpreted with caution as CHIRPP data are not nationally representative. Another study that investigated trends in service use for acute mental health before and during the first 12 months after the onset of the COVID-19 pandemic found the rate of ED visits for intentional self-injury dropped by 33% in April 2020 compared to April 2019 and had returned to near pre-pandemic levels by August in Ontario, Canada (Saunders et al. 2021).

Canada first instituted public health measures to reduce the spread of COVID-19 in March 2020. These measures intensified and were relaxed in response to each wave of the pandemic during the following 12-month period. In our study, ED visit volumes decreased for all injury types (self-harm, intentional, all ED visits) around week 11 in 2020, which corresponds to when Canada announced its first virus containment policies and public safety measures (Desson et al. 2020; Government of Canada 2021). The initial decline in ED visits was followed by a steady increase in the visit volumes for self-harm starting around week 24, which corresponds to the period when the number of new cases had stabilized at a low rate across Canada (Government of Canada 2021). The percentage change in volume for self-harm-related ED visits continued to show a positive trend after week 24 in 2020, even though COVID-19 spiked a few times later in 2020 (Government of Canada 2021). It is possible that the hesitancy and fear of seeking medical attention might have decreased over the course of pandemic, with people more comfortable presenting to hospitals with non-COVID-19-related conditions.

Internationally, there are mixed findings on how the pandemic affected self-harm-related visits to hospitals. A recent systematic review investigated the global impact of the COVID-19 pandemic on self-harm and suicidal behavior (John et al. 2020), where few studies reported a decrease in the self-harm cases (Hawton et al. 2021; Kapur et al. 2021); others reported no change or an increase during certain phases of the lockdown period during pandemic (Iob et al. 2020; McIntyre et al. 2021; Ueda et al. 2021). This variation highlights the need of continued public health surveillance to understand how the pandemic might affect the epidemiology of self-harm in Canada and across the globe. The time trend analysis in our study showed a steady decline in the percentages of self-harm ED visits during the pandemic period for both males and females, when an increase was seen in percentages of self-harm ED visits for weeks 32–52 for females, and weeks 38–42 for males during the pre-pandemic years. This steady decline during the pandemic period might be due to the interaction of the public health measures with other personal and socioeconomic factors affecting self-harm and should be further explored.

Similar to the trends reported pre-pandemic (Bethell et al. 2013; Gardner et al. 2019; Gillies et al. 2018), our study results showed higher percentages of self-harm ED visits for females when compared to males, especially for the age group 11–18 years old. Females have shown higher prevalence for self-harm across the globe in the past decade (Gardner et al. 2019; Hawton et al. 2012; McManus et al. 2014; Ohlis et al. 2020). Previous studies on the factors mediating these sex differences in self-harm found certain clinical and behavioral factors to have a higher association with females. For example, depression and anxiety were reported to have a higher association with self-harm among females than in males (Kerfoot et al. 1996; Allison et al. 2001). While the self-harm rates are higher among females than in males, both sexes have shown to have higher risk profiles for adverse outcomes (criminality, violence, substance abuse) when compared to people with no self-harm behaviors (Ohlis et al. 2020).

Another trend similar to the existing evidence (Gillies et al. 2018; Lim et al. 2019) was the higher percentages of self-harm ED visits present for adolescents (11–18 years) during pandemic when compared to pre-pandemic percentages. Since the start of the pandemic period in 2020, many cross-sectional studies on the impacts of COVID-19 and related public health measures on the mental health of children and adolescents have been published (Chadi et al. 2022). However, it is difficult to draw conclusions with no pre-pandemic comparisons in the published cross-sectional studies (Chadi et al. 2022). Limited evidence suggests there have been small increases in suicidal ideation and attempts among adolescents during pandemic compared to pre-pandemic, with possibility of differential impacts among high-risk populations (Chadi et al. 2022). Risk factors for self-harm during childhood and adolescence include being bullied and exposure to intimate partner violence at home (Lereya et al. 2013). This emphasizes the need for concrete actions to be taken to prevent injuries associated with self-harm by better identifying and addressing mental health among children and adolescents.

The methods used for self-harm were relatively unchanged during pandemic, with poisoning being the most prevalent method. A similar study reported an increase in the percentage of self-harm cases due to poisoning, but the difference was not statistically significant (McIntyre et al. 2021). Our study findings add to the current evidence of the impact of the pandemic on self-harm in Canada. However, the results should be interpreted with caution as CHIRPP is not representative of the Canadian population and although some suicides were captured in our study, CHIRPP underestimates fatalities (Crain et al. 2016). Three additional hospitals reported to CHIRPP in 2020, and their contributions to the percentages of self-harm ED visits were negligible, while the other hospitals were same across 2018, 2019, and 2020. The percentage change compared between these time periods reduces the influence of the non-representativeness on the findings except where noted previously. However, some hospitals were more or less capable of capturing the cases that did present during the pandemic, due to maintenance or a reduction in hospital staff. Other factors that might have affected the reporting of cases could be the changes in the referral patterns to the reporting hospitals or to other hospitals near the reporting hospitals. Therefore, the percentage increases found in our study might be a conservative estimate and might underestimate the true percentage change. Data analyses were done after additional time was provided to those sites for data entry. There might have been provincial differences in the percentages of self-harm ED visits; however, quantification of the provincial differences would be beyond the scope of this study and should be investigated in future studies.

## Conclusions

The current research suggests an increase in the percentages of self-harm cases among all intentional injuries in Canada for both males and females, and among adolescents aged 11–18 years in 2020 compared to 2018–2019. Females had higher percentages of self-harm than males both pre- and during the COVID-19 pandemic. The pandemic might continue to have differential influence on individuals based on age, sex, and other socioeconomic characteristics, so it is critical that self-harm-related injuries are tracked throughout the COVID-19 pandemic to provide valuable information regarding its impacts on health care and inform prevention and intervention strategies.

## Abbreviations

CHIRPP: Canadian Hospitals Injury Reporting and Prevention Program
ED: Emergency Department

## Declarations

### Availability of data and materials

Data are available from the corresponding author on reasonable request.

## References

[CR1] Allison S, Allison S, Roeger L, Martin G, Keeves J (2001). Gender differences in the relationship between depression and suicidal ideation in young adolescents. Aust N Z J Psychiatry.

[CR2] Bethell J, Bondy SJ, Lou WW, Guttmann A, Rhodes AE (2013). Emergency department presentations for self-harm among Ontario youth. Can J Public Health.

[CR3] Burstein B, Agostino H, Greenfield B (2019). Suicidal attempts and ideation among children and adolescents in US emergency departments, 2007–2015. JAMA Pediatr.

[CR4] Campeau A, Champagne AS, McFaull SR (2022). Sentinel surveillance of substance-related self-harm in Canadian emergency departments, 2011–19. BMC Public Health.

[CR5] Canner J, Giuliano K, Selvarajah S, Hammond E, Schneider E (2018). Emergency department visits for attempted suicide and self harm in the USA: 2006–2013. Epidemiol Psychiatric Sci.

[CR6] Canadian Institute for Health Information. COVID-19 Intervention Timeline in Canada. 2022. Ottawa, Canada. https://www.cihi.ca/en/covid-19-intervention-timeline-in-canada

[CR7] Canadian Institute for Health Information. Unintended consequences of COVID-19: Impact on harms caused by substance use, self-harm and accidental falls. 2021. Ottawa, Canada. https://www.cihi.ca/en/covid-19-resources/impact-of-covid-19-on-canadas-health-care-systems/unintended-consequences

[CR8] Chadi N, Ryan NC, Geoffroy MC (2022). COVID-19 and the impacts on youth mental health: emerging evidence from longitudinal studies. Can J Public Health.

[CR9] Clements C, Turnbull P, Hawton K, Geulayov G, Waters K, Ness J, Townsend E (2016). Rates of self-harm presenting to general hospitals: a comparison of data from the Multicentre Study of Self-Harm in England and Hospital Episode Statistics. BMJ Open.

[CR10] Crain J, McFaull S, Thompson W, Skinner R, Do M, Fréchette M, Mukhi S (2016). The Canadian Hospitals Injury Reporting and Prevention Program: a dynamic and innovative injury surveillance system. Health Promot Chronic Dis Prevent Can Res Policy Pract.

[CR11] Desson Z, Weller E, McMeekin P, Ammi M (2020). An analysis of the policy responses to the COVID-19 pandemic in France, Belgium, and Canada. Health Policy Technol.

[CR12] Gardner W, Pajer K, Cloutier P, Zemek R, Currie L, Hatcher S, Colman I (2019). Changing rates of self-harm and mental disorders by sex in youths presenting to Ontario emergency departments: repeated cross-sectional study. Can J Psychiatry.

[CR13] Gillies D, Christou MA, Dixon AC, Featherston OJ, Rapti I, Garcia-Anguita A, Villasis-Keever M (2018). Prevalence and characteristics of self-harm in adolescents: meta-analyses of community-based studies 1990–2015. J Am Acad Child Adolesc Psychiatry.

[CR14] Government of Canada. COVID-19 daily epidemiology update. 2021. Ottawa, Canada. https://health-infobase.canada.ca/covid-19/epidemiological-summary-covid-19-cases.html

[CR15] Griffin E, McMahon E, McNicholas F, Corcoran P, Perry IJ, Arensman E (2018). Increasing rates of self-harm among children, adolescents and young adults: a 10-year national registry study 2007–2016. Soc Psychiatry Psychiatr Epidemiol.

[CR16] Hawton K, Bergen H, Waters K, Ness J, Cooper J, Steeg S, Kapur N (2012). Epidemiology and nature of self-harm in children and adolescents: findings from the multicentre study of self-harm in England. Eur Child Adolesc Psychiatry.

[CR17] Hawton K, Casey D, Bale E, Brand F, Ness J, Waters K, Kelly S (2021). Self-harm during the early period of the COVID-19 pandemic in England: comparative trend analysis of hospital presentations. J Affect Disord.

[CR18] Holmes EA, O'Connor RC, Perry VH, Tracey I, Wessely S, Arseneault L, Ballard C (2020). Multidisciplinary research priorities for the COVID-19 pandemic: a call for action for mental health science. Lancet Psychiatry.

[CR19] Iob E, Steptoe A, Fancourt D (2020). Abuse, self-harm and suicidal ideation in the UK during the COVID-19 pandemic. Br J Psychiatry.

[CR20] Jessula S, Yanchar NL, Romao R, Green R, Asbridge M (2022). Where to start? Injury prevention priority scores for traumatic injuries in Canada. Can J Surg.

[CR21] John A, Eyles E, Webb RT, Okolie C, Schmidt L, Arensman E, Hawton K (2020). The impact of the COVID-19 pandemic on self-harm and suicidal behaviour: update of living systematic review. F1000Research.

[CR22] Johnson D, Skinner R, Cappelli M, Zemek R, McFaull S, Langill C, Cloutier P (2019). Self-Inflicted Injury-Canadian Hospitals Injury Reporting and Prevention Program (CHIRPP-SI): a new surveillance tool for detecting self-inflicted injury events in emergency departments. Can J Public Health.

[CR23] Kapur N, Clements C, Appleby L (2021). Impact of the Covid-19 pandemic on the frequency of primary care-recorded mental illness and self-harm episodes in the UK: population-based cohort study of 14 million individuals. Lancet Psychiatry.

[CR24] Keays G, Friedman D, Gagnon I (2020). Original quantitative research-Pediatric injuries in the time of COVID-19. Health Promot Chronic Dis Prevent Can Res Policy Pract.

[CR25] Kerfoot M, Dyer E, Harrington V, Woodham A, Harrington R (1996). Correlates and short-term course of self-poisoning in adolescents. Br J Psychiatry.

[CR26] Lereya ST, Winsper C, Heron J, Lewis G, Gunnell D, Fisher HL, Wolke D (2013). Being bullied during childhood and the prospective pathways to self-harm in late adolescence. J Am Acad Child Adolesc Psychiatry.

[CR27] Lim K-S, Wong CH, McIntyre RS, Wang J, Zhang Z, Tran BX, Tan W (2019). Global lifetime and 12-month prevalence of suicidal behavior, deliberate self-harm and non-suicidal self-injury in children and adolescents between 1989 and 2018: a meta-analysis. Int J Environ Res Public Health.

[CR28] Liu L, Pollock NJ, Contreras G, Tonmyr L, Thompson W (2022). Prevalence of suicidal ideation among adults in Canada: results of the second Survey on COVID-19 and mental health. Health Rep.

[CR29] McIntyre A, Tong K, McMahon E, Doherty A (2021). COVID-19 and its effect on emergency presentations to a tertiary hospital with self-harm in Ireland. Irish J Psychol Med.

[CR30] McManus S, Hassiotis A, Jenkins R, Dennis M, Aznar C, Appleby L. Suicidal thoughts, suicide attempts, and self-harm. Mental health and wellbeing in England: adult psychiatric morbidity survey. 2014:294–322.

[CR31] National Cancer Institute, Statistical Methodology and Applications Branch, Surveillance Research Program. Joinpoint Regression Program, Version 4.9.1.0 - April 2022.

[CR32] National Collaborating Centre for Mental Health. Self-harm: the short-term physical and psychological management and secondary prevention of self-harm in primary and secondary care. 2004. UK. https://www.ncbi.nlm.nih.gov/books/NBK56385/pdf/Bookshelf_NBK56385.pdf 21834185

[CR33] Ohlis A, Bjureberg J, Lichtenstein P, D’Onofrio BM, Fruzzetti AE, Cederlöf M, Hellner C (2020). Comparison of suicide risk and other outcomes among boys and girls who self-harm. Eur Child Adolesc Psychiatry.

[CR34] Public Health Agency of Canada. Canadian hospitals injury reporting and prevention program. 2020. Ottawa, Canada. https://www.canada.ca/en/public-health/services/injury-prevention/canadian-hospitals-injury-reporting-prevention-program.html

[CR35] Ruch DA, Heck KM, Sheftall AH, Fontanella CA, Stevens J, Zhu M, Horowitz LM (2021). Characteristics and precipitating circumstances of suicide among children aged 5 to 11 years in the United States, 2013–2017. JAMA Netw Open.

[CR36] Saunders NR, Toulany A, Deb B, Strauss R, Vigod SN, Guttmann A, Chiu M (2021). Acute mental health service use following onset of the COVID-19 pandemic in Ontario, Canada: a trend analysis. Can Med Assoc Open Access J.

[CR37] Skinner R, McFaull S, Draca J, Frechette M, Kaur J, Pearson C, Thompson W (2016). Suicide and self-inflicted injury hospitalizations in Canada (1979 to 2014/15). Health Promot Chronic Dis Prevent Can Res Policy Pract.

[CR38] Skinner R, Irvine B, Branchard B, Williams G, Pearson C, Kaur J, Yao X, Merklinger L, Lary T (2017). A contextual analysis of the Suicide Surveillance Indicators. Health Promot Chronic Dis Prevent Can Res Policy Pract.

[CR39] Stewart C, Crawford PM, Simon GE (2017). Changes in coding of suicide attempts or self-harm with transition from ICD-9 to ICD-10. Psychiatr Serv.

[CR40] Ueda M, Nordström R, Matsubayashi T (2021). Suicide and mental health during the COVID-19 pandemic in Japan. J Public Health (oxford, England).

[CR41] Vigo D, Jones L, Thornicroft G, Atun R (2020). Burden of mental, neurological, substance use disorders and self-harm in North America: a comparative epidemiology of Canada, Mexico, and the United States. Can J Psychiatry.

[CR42] World Health Organization. Public health action for the prevention of suicide: a framework. 2012. Geneva, Swtizerland. https://apps.who.int/iris/bitstream/handle/10665/75166/?sequence=1

[CR43] World Health Organization. Preventing Suicide: a Global Imperative. 2014. Geneva, Swtizerland. https://www.who.int/publications/i/item/9789241564779

